# Puerarin alleviates osteoporosis in the ovariectomy-induced mice by suppressing osteoclastogenesis via inhibition of TRAF6/ROS-dependent MAPK/NF-κB signaling pathways

**DOI:** 10.18632/aging.103976

**Published:** 2020-11-07

**Authors:** Long Xiao, Mengdan Zhong, Yu Huang, Jie Zhu, Wenkai Tang, Danyong Li, Jiandong Shi, Aiqing Lu, Huilin Yang, Dechun Geng, Hong Li, Zhirong Wang

**Affiliations:** 1 Department of Orthopedics, Zhangjiagang TCM Hospital Affiliated to Nanjing University of Chinese Medicine, Zhangjiagang 215600, China; 2 Department of Orthopedics, The First Affiliated Hospital of Soochow University, Suzhou 215006, China; 3 Department of Endocrinology, Zhangjiagang TCM Hospital Affiliated to Nanjing University of Chinese Medicine, Zhangjiagang 215600, China; 4 Department of Gynecology, The First Affiliated Hospital of Soochow University, Suzhou 215006, China; 5 Department of Gynecology, The First People's Hospital of Zhangjiagang, Soochow University, Zhangjiagang 215600, China

**Keywords:** puerarin, osteoclast, reactive oxygen species, osteoporosis

## Abstract

In this study, we investigated the mechanisms by which puerarin alleviates osteoclast-related loss of bone mass in ovariectomy (OVX)-induced osteoporosis model mice. Puerarin-treated OVX mice exhibited higher bone density, fewer tartrate-resistant acid phosphatase (TRAcP)-positive osteoclasts, and levels of lower reactive oxygen species (ROS) within bone tissues than vehicle-treated OVX mice. Puerarin suppressed *in vitro* osteoclast differentiation, hydroxyapatite resorption activity, and expression of osteoclastogenesis-related genes, such as NFATc1, MMP9, CTSK, Acp5 and c-Fos, in RANKL-induced bone marrow macrophages (BMMs) and RAW264.7 cells. It also reduced intracellular ROS levels by suppressing expression of TRAF6 and NADPH oxidase 1 (NOX1) and increasing expression of antioxidant enzymes such as heme oxygenase-1 (HO-1). Puerarin inhibited TRAF6/ROS-dependent activation of the MAPK and NF-κB signaling pathways in RANKL-induced RAW264.7 cells, and these effects were partially reversed by HO-1 silencing or TRAF6 overexpression. These findings suggest puerarin alleviates loss of bone mass in the OVX-model mice by suppressing osteoclastogenesis via inhibition of the TRAF6/ROS-dependent MAPK/NF-κB signaling pathway.

## INTRODUCTION

Osteoporosis is a progressively degenerative bone disease associated with aging and is characterized by reduced bone mass and structural deterioration of the bone tissue, resulting in weak and fragile bones with increased risk of fractures. The dysfunctional activities of the osteoblasts and osteoclasts are the primary cause of osteoporosis [1]. In the postmenopausal women, estrogen deficiency causes osteoporosis because of excessive osteoclast activity resulting in reduced bone mass and decreased bone strength [2–4]. Hence, inhibition of osteoclast-induced bone resorption is the main therapeutic strategy for patients with osteopenia and osteoporosis.

Several studies have shown that osteoporosis is associated with increased oxidative stress [5, 6]. The serum antioxidant levels are significantly reduced in the patients with osteoporosis [7–10]. The levels of oxidative stress-related biomarkers are significantly higher and the antioxidant levels are significantly lower in the serum of postmenopausal women with osteoporosis. Furthermore, estrogen withdrawal weakens the bone defense mechanisms against oxidative stress and increases the risk of osteoporotic damage [11]. Osteoclasts are giant polykaryotic cells that are derived from the mononuclear macrophage lineage of the blood cells and are generated by the fusion of mononuclear progenitor cells [12]. Previous studies have shown that bone resorption activity of the osteoclasts is modulated by the reactive oxygen species (ROS) levels [13–16].

Puerarin is a major isoflavone glycoside extracted from the Chinese herb *Pueraria radix*. It has shown immense therapeutic potential for reducing blood pressure [17, 18], blood sugar [19, 20], blood lipids [21, 22], and cancer [23–25]. Puerarin decreases bone mass loss (osteopenia) in a concentration dependent manner by stimulating osteoprotegerin (OPG), which promotes osteogenic-specific transcription factors and inhibits Receptor Activator of Nuclear factor-κB (NF-κB) Ligand (RANKL)—a key promoter of osteoclastogenesis [26–28]. Several studies also show that puerarin inhibits osteoclastogenesis and bone resorption. For example, a recent study demonstrated that puerarin inhibits osteoclastogenesis by inhibiting RANKL-dependent and RANKL-independent autophagic responses [29]. However, the mechanism through which puerarin inhibits osteoclastogenesis and osteoporosis are unclear. Therefore, in this study, we investigated the mechanism through which puerarin inhibits osteoclastogenesis and osteoporosis using *in vitro* cell models and the *in vivo* ovariectomy (OVX)-induced osteoporosis model mice.

## RESULTS

### Puerarin protects against bone mass loss in the OVX mouse model

**Figure 1:**
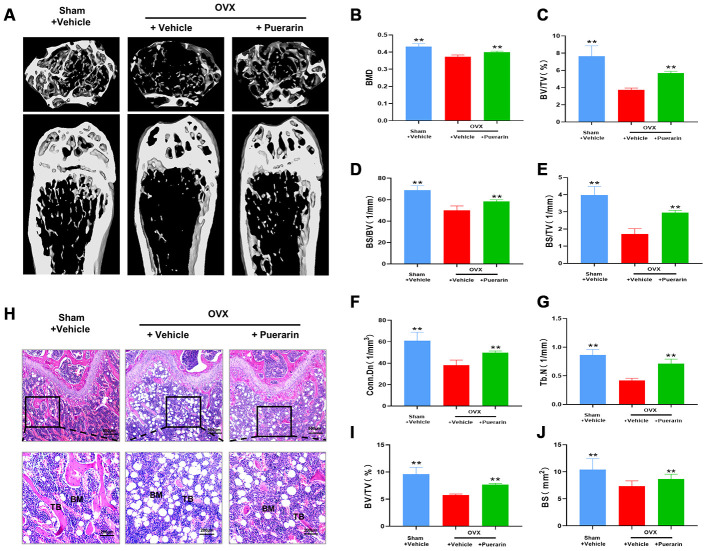
**Puerarin protects against bone mass loss in the *in vivo* OVX-induced osteoporosis model mice.** (**A**) Representative 3D- μCT images of the femur bone in the sham, OVX+vehicle, and OVX+puerarin group mice. (**B**–**G**) Comparative analysis of the bone structural parameters such as bone mass density (BMD in g/mm^3^), ratio of bone volume to total volume (BV/TV in %), ratio of bone surface to bone volume (BS/BV in 1/mm), ratio of bone surface to total volume (BS/TV in 1/mm), connectivity density (Conn.Dn in 1/mm) and trabecular number (Tb.N in 1/mm^2^)in the sham, OVX+vehicle, and OVX+puerarin group mice (n=5 per group). Note: **P <0.01 vs. OVX+vehicle group. (**H**) Representative H&E stained femur bone sections of the sham, OVX+vehicle, and OVX+puerarin group mice (n=5 per group). (**I**–**J**) The analysis of the bone parameters, BV/TV and BS based on the H&E staining sections of the femur bone from the sham, OVX+vehicle, and OVX+puerarin group mice (n=5 per group). Note: **P <0.01 vs. the OVX+vehicle group.

We performed 3D-μCT analysis to evaluate the bone structural features in the sham, OVX and OVX+puerarin group mice. Overall, the results showed that bone mass loss was significantly higher in the OVX group mice compared to the sham group, but significantly reduced in the OVX+puerarin group (Figure 1A). As shown in Figure 1B–1G, the various bone structural parameters based on the 3D-μCT analysis for the sham, OVX, and OVX+puerarin mice (n=5) were as follows: BMD in g/cm^3^ (0.432±0.017 vs. 0.372±0.011 vs. 0.399±0.006), BV/TV in % (7.62±1.22 vs. 3.74±0.20 vs. 5.68±0.22), BS/BV in 1/mm (68.87±4.14 vs. 50.13±4.05 vs. 58.32±1.60), BS/TV in 1/mm (3.99±0.49 vs. 1.70±0.32 vs. 2.96±0.12), Conn.Dn in 1/mm^2^ (60.90±8.01 vs. 38.28±4.66 vs. 49.90±1.49), Tb.N in l/mm (0.86±0.10 vs. 0.42±0.04 vs. 0.71±0.08). H&E staining of the femur bone tissue sections from the sham, OVX and OVX+puerarin group mice confirmed that puerarin treatment decreased OVX-induced bone mass loss (Figure 1H). H&E staining results showed that the bone surface (BS) and bone volume (BV) were significantly reduced in the OVX group compared to the sham group, but were significantly higher in the OVX+puerarin group compared to the OVX group (Figure 1G and 1I). These results showed that puerarin significantly reduces bone mass loss in the OVX-induced osteoporosis model mice.

**Figure 2:**
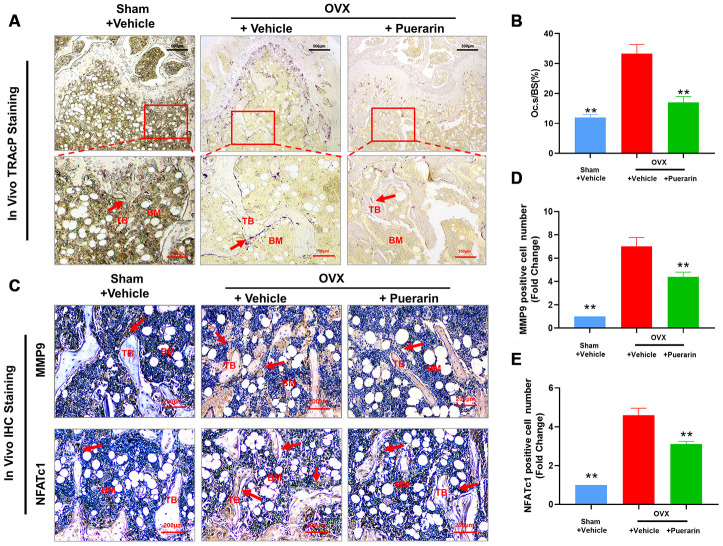
**Puerarin treatment reduces the numbers of osteoclasts in the bone tissue of OVX-induced osteoporosis model mice.** (**A**, **B**) Representative images show the tartrate-resistant acid phosphatase (TRAcP) activity staining in the bone sections from the sham, OVX+vehicle, and OVX+puerarin mice. Also shown is the estimation of the osteoclast surface relative to the bone surface (Oc.S/BS) in the femur bones of the sham, OVX+vehicle, and OVX+puerarin group mice (n=5 per group). Note: **P <0.01 vs. the OVX+vehicle group. (**C**–**E**) Representative images show the IHC staining of the femur bone sections from the sham, OVX+vehicle, and OVX+puerarin mice with antibodies against bone resorption markers, MMP9 and NFATc1. Also shown is the quantitative analysis of MMP9- and NFATc1-postive cells in the femur bone sections from the sham, OVX+vehicle, and OVX+puerarin mice (n=5 per group). Note: **P <0.01 vs. the OVX+vehicle group.

Several studies have implicated excessive activity of the osteoclasts in the pathogenesis related to osteoporosis. Therefore, we measured the effect of puerarin on the osteoclast numbers and function in the *in vivo* OVX-induced osteoporosis model mice. Tartrate resistant Acid Phosphatase (TRAcP) activity staining of the bone sections demonstrated that the numbers of osteoclasts in the bone tissues of the OVX+ puerarin group mice were significantly lower compared to those in the OVX group mice (Figure 2A). Puerarin treatment significantly reduced the numbers of osteoclasts and the surface area of the osteoclasts on the bone surface compared to the OVX group (Figure 2B). IHC analysis of bone resorption markers MMP9 and NFATc1 showed that the numbers of MMP9- and NFATc1-positive cells were significantly higher in the OVX group compared to the sham group, but were significantly reduced in the OVX+ puerarin group compared to the OVX group (Figure 2C–2E). Moreover, we did not observe any adverse events during the OVX procedure or puerarin treatment. The H&E staining of the liver and kidney tissues in the OVX+puerarin treated mice showed normal morphology (Supplementary Figure 1). Overall, these results demonstrate that puerarin reduces bone loss in the OVX-induced osteoporosis mice by suppressing the proliferation of TRAcP-positive osteoclasts and inhibiting the bone resorption activity of the osteoclasts.

### Puerarin reduces ROS levels in OVX-induced osteoporosis model mice

**Figure 3:**
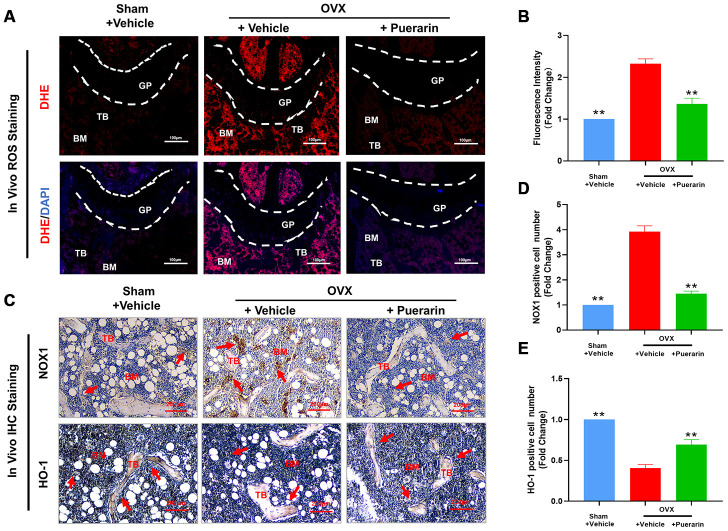
**Puerarin treatment reduces ROS levels in the bone tissues of the OVX-induced osteoporosis model mice.** (**A**, **B**) Fluorescence images show the ROS levels based on DHE staining in the cryosections of femur bone tissues from the sham, OVX+vehicle, and OVX+puerarin mice (n=5 per group). Note: **P <0.01 vs. the OVX+vehicle group. (**C**–**E**) Representative images show the IHC staining of the femur bone sections from the sham, OVX+vehicle, and OVX+puerarin mice with antibodies against NOX1 and HO-1. Also shown is the quantitative analysis of NOX1 and HO-1 expression in the bone sections from the sham, OVX+vehicle, and OVX+puerarin mice (n=5 per group). Note: **P <0.01 vs. the OVX+vehicle group.

Osteoclast differentiation involves increased ROS levels and activation of redox signaling pathways [30]. Therefore, we investigated *in vivo* ROS levels by dihydroethidium (DHE) staining of the cryosectioned femur bone tissues from the three mouse groups. DHE is a redox-sensitive fluorescent dye that is taken up by living cells and oxidized by superoxide anions to produce ethidium^+^, which binds to RNA and DNA to produce red fluorescence that can be estimated. ROS levels were significantly higher in the bone tissues of the OVX group mice compared to the sham group, but were reduced in the OVX+puerarin group (Figure 3A and 3B). Furthermore, IHC analysis showed that NOX1 (NADH oxidase 1; generates ROS or oxidative role) protein levels were higher and HO-1 (Heme Oxygenase 1; removes ROS or antioxidant role) protein levels were reduced in the bone tissues of the OVX group mice compared to the sham group, but, NOX1 levels were reduced and HO-1 levels were increased in the bone tissues of the OVX+puerarin group mice (Figure 3C–3E). These data suggest that puerarin suppresses ROS levels in the OVX-induced osteoporosis mice by increasing levels of antioxidant enzymes such as HO-1 and decreasing levels of oxidant enzymes such as NOX-1.

### Puerarin inhibits *in vitro* osteoclast differentiation

**Figure 4:**
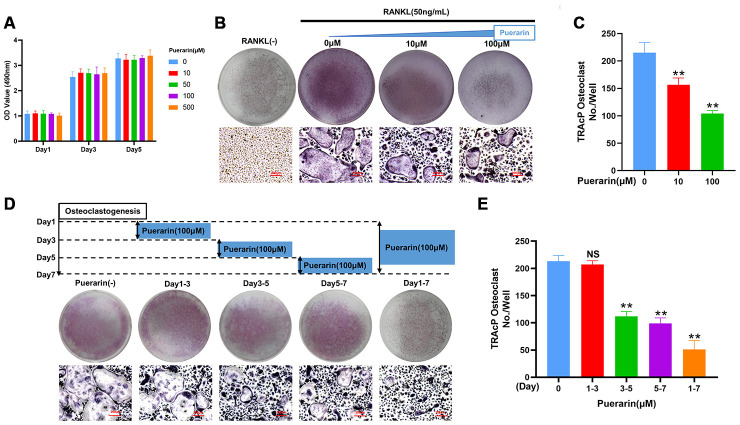
**Puerarin inhibits *in vitro* osteoclastogenesis.** (**A**) CCK-8 assay results show the viability of BMMs treated with 0, 10, 50, 100 and 500 μM puerarin (n=5 per group). (**B**–**C**) TRAcP assay analysis shows the numbers of TRAcP-positive cells (>3 nuclei) in BMMs treated with 50 ng/mL M-CSF, 50 ng/mL RANKL, and 0, 10, or 100 μM puerarin. Note: n=3 per group; **P <0.01 vs. the control group. (**D**, **E**) TRAcP assay analysis shows the numbers of TRAcP-positive cells in BMMs stimulated with 50 ng/mL M-CSF, 50 ng/mL RANKL and 100 μM puerarin for the indicated days during osteoclastogenesis, Note: n=3 per group, NS: Not statistically significant, **P <0.01 vs. the control group (without puerarin treatment).

CCK-8 assays demonstrated that treatment with 10 and 100 μM puerarin did not reduce BMM viability (Figure 4A). The *in vitro* osteoclast differentiation was evaluated by treating BMMs with 50 ng/mL M-CSF and 50 ng/mL RANKL in the presence of 0, 10, and 100 μM puerarin. The results showed that puerarin decreased the formation of multinucleated osteoclasts in a dose-dependent manner compared to the RANKL-induced group (Figure 4B–4C). Furthermore, the number of TRAcP-positive osteoclasts decreased when RANKL-induced BMMs were treated with 100 μM puerarin on days 3-7 but had no effects when treated on days 1-3 (Figure 4D, 4E).

### Puerarin affects *in vitro* hydroxyapatite resorption by osteoclasts

**Figure 5:**
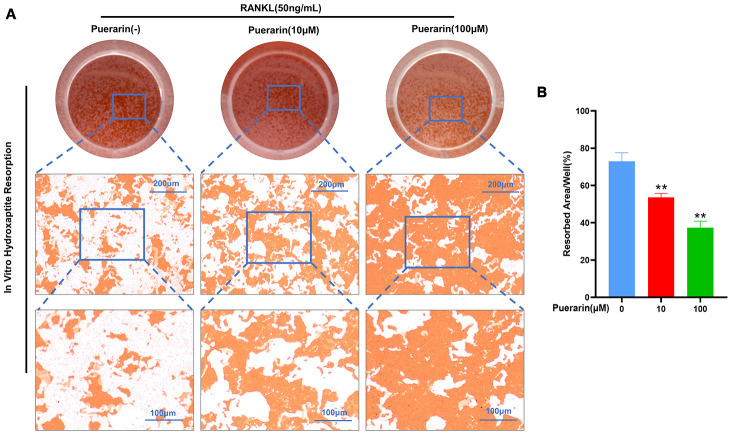
**Puerarin suppresses *in vitro* hydroxyapatite resorption activity of the osteoclasts.** (**A**) Representative images show the total numbers of osteoclasts and the resorption pits formed in the hydroxyapatite resorption assay in the BMMs treated with 50 ng/mL M-CSF, 50 ng/mL RANKL and 0, 10, or 100 μM puerarin. (**B**) Hydroxyapatite resorption assay analysis showsthe total resorbed hydroxyapatite area in the BMMs treated with 50 ng/mL M-CSF, 50 ng/mL RANKL and 0, 10, or 100 μM puerarin. Note: n=3 per group; **P <0.01 vs. the control group (without puerarin treatment).

We next analyzed the effects of puerarin on osteoclast resorptive activity using the hydroxyapatite resorption assay. The BMMs induced with 50 ng/mL M-CSF and 50 ng/mL RANKL generated numerous resorption pits, but, puerarin treatment significantly reduced the numbers of osteoclasts and the resorption pits in a dose-dependent manner (Figure 5A). We observed resorption pits in nearly 73% of the hydroxyapatite surface area in the RANKL-induced group compared to 54% and 37% in the BMMs treated with 10μM and 100μM puerarin, respectively (Figure 5B). These results demonstrate that puerarin inhibits *in vitro* resorption activity of osteoclasts in a dose-dependent manner.

### Puerarin inhibits formation of the F-actin podosome ring on the surface of the osteoclasts and the expression of osteoclastogenesis-related genes

**Figure 6:**
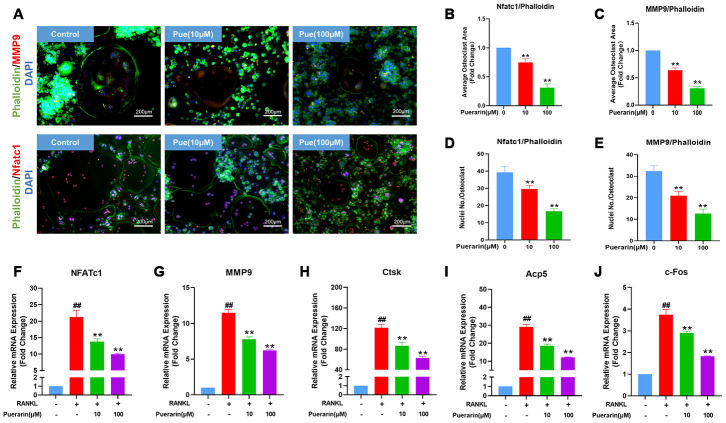
**Puerarin reduces F-actin ring formation in the osteoclasts and suppresses osteoclastogenesis-related gene expression.** (**A**) Representative images show the podosome belt formation in the osteoclasts formed from the RANKL and RANKL+ indicated concentrations of puerarin-treated BMMs. The cells were stained with antibodies against MMP9/NFATc1 (red), phalloidin for F-actin (green) and the nuclei were stained with DAPI (blue). (**B**, **C**) Quantitative analysis of the F-actin ring area in the osteoclasts from the RANKL and RANKL+ indicated concentrations of puerarin-treated BMMs. Note: n=3 per group, ** *P <*0.01 vs. the control group (without puerarin treatment). (**D**, **E**) Quantitative analysis of the numbers of nuclei per osteoclast in the control BMMs and BMMs induced the RANKL and RANKL+ indicated concentrations of puerarin-treated. Note: n=3 per group; ** *P <*0.01 vs. the control group (without puerarin treatment). (**F–J**) QRT-PCR analysis of the mRNA levels of osteoclastogenesis-related genes, NFATc1, MMP9, CTSK, Acp5, and c-Fos, in the control BMMs and BMMs induced with RANKL and RANKL+ indicated concentrations of puerarin. Note: n=3 per group; ^##^ *P <*0.01 vs. the control group (without RANKL induce and puerarin treatment); ** *P <*0.01 vs. the RANKL-induced group (without puerarin treatment).

The sealing zone formed around the mature osteoclasts consists of a ring of filamentous actin (F-actin) with clear margins and this process is required for bone resorption [31]. Therefore, we analyzed the effects of puerarin on the F-actin ring formation and bone resorption by co-staining with phalloidin and antibodies against two critical osteoclastogenesis-specific proteins, MMP9 and NFATc1. The RANKL-induced BMMs showed well-defined F-actin sealing rings with intact nuclei and strongly positive MMP9 and NFATc1 expression, but, puerarin reduced the size of the rings, number of nuclei, and the expression of MMP9 and NFATc1 in a concentration-dependent manner (Figure 6A–6E).

Osteoclast differentiation is related to the expression levels of the osteoclast-specific genes such as NFATc1, MMP9, CTSK, Acp5 and c-Fos [32, 33]. QRT-PCR analysis showed that the relative mRNA expression of NFATc1, MMP9, CTSK, Acp5 and c-Fos was significantly upregulated in the RANKL-induced BMMs, but significantly reduced by puerarin in a concentration-dependent manner (Figure 6F–6J). Collectively, these results demonstrate that puerarin inhibits *in vitro* osteoclastogenesis by suppressing the expression of specific osteoclast differentiation genes.

### Puerarin suppresses ROS levels by enhancing the expression of antioxidant enzymes

**Figure 7:**
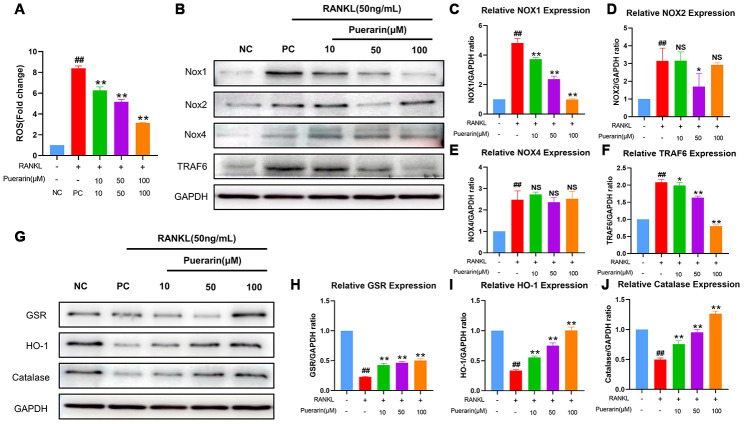
**Puerarin inhibits ROS by upregulating antioxidant enzymes in the RANKL-induced RAW264.7 cells.** (**A**) Flow cytometry analysis of ROS levels using DCFH2-DA in the untreated, RANKL-treated and indicated concentrations of puerarin+RANKL-treated RAW264.7 cells. Note: n=3 per group; ^##^ *P <*0.01 vs. the untreated group; ** *P <*0.01 vs. the RANKL-treated group. (**B**) Representative western blot images show the levels of NOX1/2/4 and TRAF6 proteins in the untreated, RANKL-induced and indicated concentrations of puerarin+RANKL treated RAW264.7 cells. (**C**–**F**) The histogram shows the relative levels of NOX1/2/4 and TRAF6 proteins in the untreated, RANKL- and RANKL+ indicated concentrations of puerarin-treated RAW264.7 cells. Note: n=3 per group; NS: Not statistically significant, ^##^ *P <*0.01 vs. the untreated group; * *P <*0.05, ** *P <*0.01 vs. the RANKL-induced group. (**G**) Representative western blot images show the levels of antioxidant enzymes, GSR, HO-1, and catalase in the untreated, RANKL- and RANKL+ indicated concentrations of puerarin-treated RAW264.7 cells. (**H**–**J**) The histogram shows the relative levels of GSR, HO-1 and catalase proteins in the untreated, RANKL- and RANKL+ indicated concentrations of puerarin-treated RAW264.7 cells. Note: n=3 per group; ^##^ *P <*0.01 vs. the untreated group; ** *P <*0.01 vs. the RANKL-induced group.

We then performed flow cytometry analysis to determine the effects of puerarin on intracellular ROS levels during RANKL-induced osteoclastogenesis using the ROS-sensitive dye, DCFH-DA. Puerarin reduced ROS levels in a dose-dependent manner in the RANKL-induced RAW264.7 cells (Figure 7A).

The NADPH oxidases (NOXs), namely, NOX1, NOX2 and NOX4, are important contributors of cellular ROS [34]. Moreover, the levels of antioxidant enzymes, glutathione disulfide reductase (GSR), HO-1, and catalase, play a crucial role in downregulating cellular ROS [35, 36]. Therefore, we analyzed the effects of puerarin on the levels of NOX1, GSR, HO-1 and catalase proteins during *in vitro* osteoclastogenesis. The NOX1 protein levels were significantly upregulated in the RANKL-induced RAW264.7 cells, but were reduced by puerarin in a dose-dependent manner (Figure 7B, 7C). Furthermore, GSR, HO-1 and catalase levels were reduced in the RANKL-induced RAW264.7 cells, but were upregulated by puerarin in a dose dependent manner (Figure 7G–7J).

**Figure 8:**
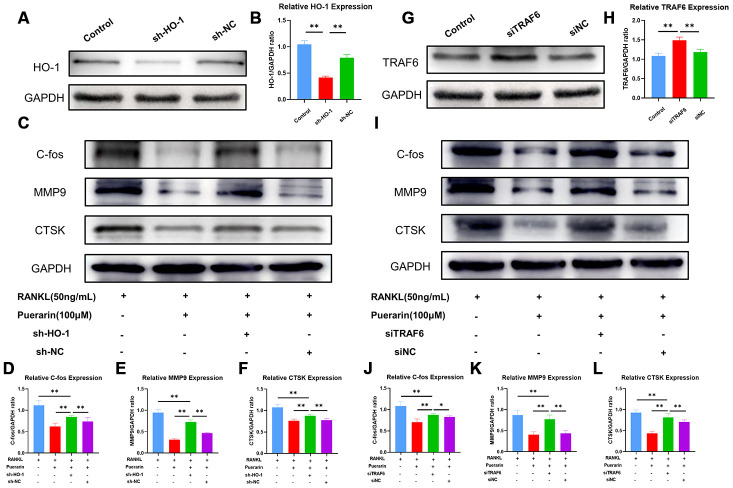
**Puerarin inhibits ROS levels by upregulating HO-1 and downregulating TRAF6.** (**A**) Representative western blot images show the HO-1 protein levels in the control and HO-1 knockdown RAW264.7 cells. (**B**) The histogram plots show the relative HO-1 levels in the control and HO-1 knockdown RAW264.7 cells. Note: n=3 per group; **P <0.01. (**C**) Representative western blots show the levels of C-fos, MMP9 and CTSK in the control and HO-1 knockdown RAW264.7 cells treated with RANKL or RANKL+puerarin. (**D**–**F**) The histogram plots show the levels of C-fos, MMP9 and CTSK proteins in the control and HO-1 knockdown, RAW264.7 cells treated with RANKL or RANKL+puerarin. Note: n=3 per group, **P <0.01. (**G**) Western blot analysis shows the TRAF6 protein levels in the control and TRAF6 overexpressing RAW264.7 cells. (**H**) The histogram plots show the relative TRAF6 protein levels in the control and TRAF6 overexpressing RAW264.7 cells. Note: n=3 per group; **P <0.01. (**I**) Representative western blots show the levels of C-fos, MMP9 and CTSK in the control and TRAF6 overexpressing RAW264.7 cells treated with RANKL or RANKL+puerarin (**J**–**L**) The histogram plots show the levels of C-fos, MMP9 and CTSK proteins in the control and TRAF6 overexpressing RAW264.7 cells treated with RANKL or RANKL+puerarin. Note: n=3 per group; *P <0.05, **P <0.01.

Next, we analyzed the effects of puerarin on ROS signaling by lentiviral knockdown of HO-1 (Figure 8A and 8B) and overexpression of TRAF6 (Figure 8G and 8H) in the RAW264.7 cells. As shown in Supplementary Figure 2A, ROS levels were significantly reduced in the puerarin+RANKL-induced RAW264.7 cells compared to the RANKL-induced RAW264.7 cells, but, the effects of puerarin were abolished by HO-1 knockdown or TRAF6 overexpression. Furthermore, the levels of osteoclast differentiation-related proteins, c-Fos, MMP9 and CTSK were significantly downregulated in the puerarin+RANKL-induced RAW264.7 cells compared to the RANKL-induced RAW264.7 cells, but these effects were partially abrogated by HO-1 knockdown (Figure 8C–8F) and TRAF6 overexpression (Figure 8I, 8L). These results were further confirmed by the TRAcP staining and hydroxyapatite resorption assays (Supplementary Figure 2B–2E). Taken together, these results suggest that puerarin partly suppresses ROS production by inhibiting Nox1 and TRAF6 expression and increasing the levels of antioxidant enzymes such as HO-1.

### Puerarin suppresses RANKL-induced osteoclastogenesis by inhibiting the NF-κB and MAPK signaling pathways

**Figure 9:**
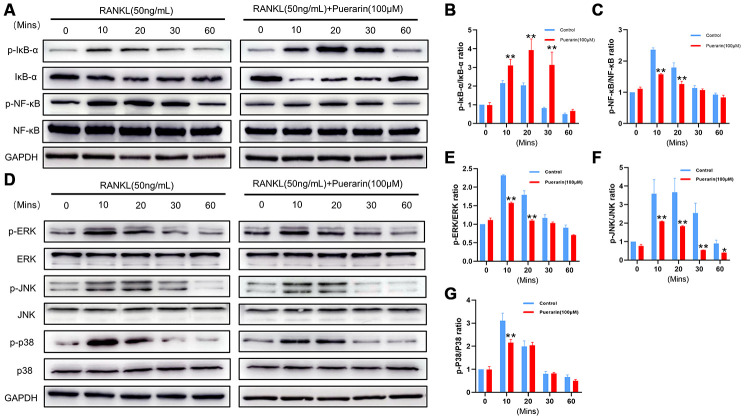
**Puerarin inhibits RANKL-induced osteoclast differentiation by suppressing the NF-κB and MAPK signaling pathways.** (**A**) Representative western blot images show the levels of p-IκBα, IκBα, NF-κB-p65, and p-NF-κB-p65 at 0, 10, 20, 30 and 60 mins in the RANKL- andRANKL+puerarin-treated RAW264.7 cells. (**B**–**C**) The histograms show the relative levels of p-IκBα and p-NF-κB proteins at 0, 10, 20, 30 and 60 min in the RANKL-, and RANKL+puerarin-treated RAW264.7 cells. Note: n=3 per group; *P <0.05 **P <0.01 vs. the RANKL-induced group. (**D**) Representative western blot images show the levels of ERK, p-ERK, JNK, p-JNK, P38 and p-P38 MAP kinases at 0, 10, 20, 30 and 60 min in the RANKL-, and RANKL+puerarin-treated RAW264.7 cells. (**E**–**G**) The histograms show the relative levels of the p-ERK, p-JNK, and p-p38 proteins at 0, 10, 20, 30 and 60 min in the RANKL-, and RANKL+puerarin-treated RAW264.7 cells. Note: n=3 per group, *P <0.05, **P <0.01 vs. the RANKL-induced group at the same time point.

The binding of RANKL to its receptor, RANK recruits TRAF6 to its cytoplasmic domain and subsequently activates the MAPK and NF-κB signaling pathways, which then induce the expression of osteoclastogenesis genes [37]. The expression of the adaptor protein TRAF6 was significantly increased in the RANKL-induced RAW264.7 cells, but was reduced in the puerarin+ RANKL-induced RAW264.7 cells (Figure 7B and 7F). Next, we examined the effects of puerarin on the activation of NF-κB and MAPK signaling pathways. Western blotting analysis showed that phospho-NF-κB-p65 and phospho-IκB-α levels were significantly higher in the RANKL-treated RAW264.7 cells, whereas, puerarin treatment decreased the levels of phospho-NF-κB-p65 and further increased phospho-IκB-α levels (Figure 9A–9C). We then analyzed the phosphorylation status of ERK, JNK and p38 MAP Kinases by western blotting. The phospho-ERK, phospho-JNK and phospho-p38 levels were significantly higher in the RANKL-induced RAW264.7 cells, but were reduced by puerarin in a time-dependent manner (Figure 9D–9G). These data suggest that puerarin inhibits osteoclast differentiation by suppressing TRAF6 expression and activation of the NF-κB and MAPK pathways.

## DISCUSSION

Osteoporosis is a progressive bone disease that affects a significant number of people worldwide, especially the aging populations, and is characterized by bone fragility and increased risk of fractures; it is associated with high morbidity, mortality, and health care costs [38, 39]. Postmenopausal osteoporosis typically occurs about 5-10 years after menopause because of decreasing estrogen levels [40, 41]. However, long-term preventive and curative medical care is not currently available. Estrogen is used primarily for the short-term prevention of osteoporosis but is not preferred for the long-term treatment because of side effects including cardiovascular problems and breast cancer risk [42]. Bisphosphonates are the most commonly used drugs for the prevention and treatment of osteoporosis, but their long-term use is not recommended because of side effects including muscle and joint pain due to unknown mechanisms and osteonecrosis of the jaw [43]. Teriparatide is a novel and promising drug to treat osteoporosis, but its use is limited to 18-24 months because of a high incidence of hypercalcemia [44]. Therefore, alternative and complementary long-term therapies with fewer side effects are urgently required for the treatment of clinical postmenopausal osteoporosis. In this study, we demonstrate the potential benefits of puerarin for the treatment of clinical postmenopausal osteoporosis. We demonstrate that puerarin decreases bone mass loss by inhibiting osteoclast differentiation and activity through reduction of ROS levels and ROS-dependent MAPK and NF-κB signaling pathways in the OVX-induced osteoporosis model mice.

Oxidative stress plays a pivotal role in the pathogenesis of osteoporosis [32, 45, 46]. Osteoporosis involves imbalance between oxidation mechanisms that generate ROS and the antioxidant mechanisms that scavenge ROS. Hence, antioxidant therapy has been investigated as a viable therapy for patients with osteoporosis [47, 48]. However, there is no consensus on the clinically relevant oxidative stress-related biomarkers for osteoporosis. Direct estimation of ROS levels is difficult because of their instability and short half-life. Therefore, oxidative stress is generally assessed indirectly by measuring the activity of antioxidant enzymes [49–51]. Sendur et al. reported that the serum levels of antioxidant enzymes are higher in the postmenopausal women with osteoporosis [52]. In this study, we measured the *in vivo* levels of ROS levels using the fluorescent ROS-sensitive dye, DHE, which has been used to detect ROS levels in the dissected aorta and renal arteries [53]. *In vivo* DHE assay demonstrated that puerarin decreases ROS levels in the bone marrow of OVX-induced osteoporosis model mice. Furthermore, puerarin treatment significantly reduces loss of bone mass in the OVX-induced osteoporosis model mice. This suggests that puerarin inhibits oxidative stress and suppresses osteoclast differentiation and function.

Osteoclasts play a significant role in bone resorption and bone remodeling [54]. Hence, osteoclasts are considered as therapeutic targets for bone-related diseases including osteoporosis. Bisphosphonates are drugs that inhibit the activity of osteoclasts and are commonly used to treat osteoporosis [55]. However, bisphosphonates are not effective in all individuals. Our study demonstrates that puerarin inhibits osteoclast differentiation and activity by reducing the ROS levels. Previous studies have shown that ROS play a key role in the differentiation and survival of osteoclasts. For example, Ha et al. demonstrated that RANKL stimulation induces ROS production and activates downstream signaling pathways that mediate osteoclastogenesis [56]. Our results show that puerarin suppresses differentiation of BMMs and RAW264.7 cells into osteoclasts by reducing the ROS levels.

Autophagy plays a crucial role in the intricately orchestrated process of osteoclastic differentiation [57]. Recently, Zhang et al showed that puerarin prevents RANKL-induced osteoclastogenesis by inhibiting RANKL-dependent autophagy [29]. In our study, we observed that RANKL stimulation upregulated autophagy, but puerarin reduced the expression of the autophagy protein, LC3B (Supplementary Figure 3D, 3E), and the autophagic vesicles as determined by MDC staining (Supplementary Figure 3C). These results demonstrate that puerarin reduces autophagy in the osteoclasts, thereby affecting their activity in osteoporosis.

Furthermore, we investigated the mechanism of osteoclast differentiation and function in regard to ROS and the NF-κB and MAPK pathways. RANKL induction increased the levels of intracellular ROS accompanied by the activation of NF-κB and MAPK signaling pathways, but these effects were significantly reduced by puerarin. Binding of RANKL to its receptor, RANK, recruits TRAF6, an adaptor molecule, which in turn activates MAPKs, NF-κB, and NFATc1 [58]. This cascade represents a key step in RANKL-induced osteoclast differentiation. The classic NF-κB pathway involves the phosphorylation and subsequent degradation of IκBα, which allows nuclear translocation of the p65 subunit of NF-κB and NF-κB-dependent gene expression. Furthermore, MAPK pathway activation involves the phosphorylation of p38, ERK1/2, and JNK. Several studies have demonstrated that the MAPK signaling pathway is involved in NF-kB activation [59–61]. While TRAF6 does not directly participate in ROS production, the dominant-mutant form of TRAF6 decreases intracellular ROS levels [58]. Zhou et al. reported that RANKL stimulation also increases the levels of intracellular ROS [62]. Furthermore, activation of ROS/MAPK/NF-κB signaling cascade plays a crucial role in hyperactivation of osteoclasts in osteoporosis [63]. Antioxidant enzymes also play a crucial role in modulating bone homeostasis and prevent osteoclast-mediated bone mass loss by reducing the ROS levels [64, 65]. In the present study, we demonstrate that puerarin treatment significantly reduces the ROS/MAPK/NF-κB signaling pathway, which is required for osteoclast differentiation and function.

Finally, this study has some limitations. Firstly, we demonstrate that puerarin inhibits osteoclastogenesis, both *in vivo* and *in vitro*, but, the osteoblasts also play a significant role in the bone homeostasis. We observe that puerarin promotes *in vitro* osteoblast differentiation (Supplementary Figure 4) by activating the p38 MAPK and downregulating the NF-κB signaling pathway in a dose-dependent manner (Supplementary Figure 5). However, the effects of puerarin on osteoblastogenesis and osteoporosis need further investigations. Secondly, in this study, we demonstrate the beneficial effects of puerarin in the OVX-induced osteoporosis model mice, but, its effects on other experimental osteoporosis models remains to be investigated. Thirdly, pathogenesis of osteoporosis involves immune dysfunction and systemic inflammation. However, the effects of puerarin are not known regarding these aspects. Hence, future investigations are required to discover the effects of puerarin on inflammation and osteoporosis.

**Scheme 1.**
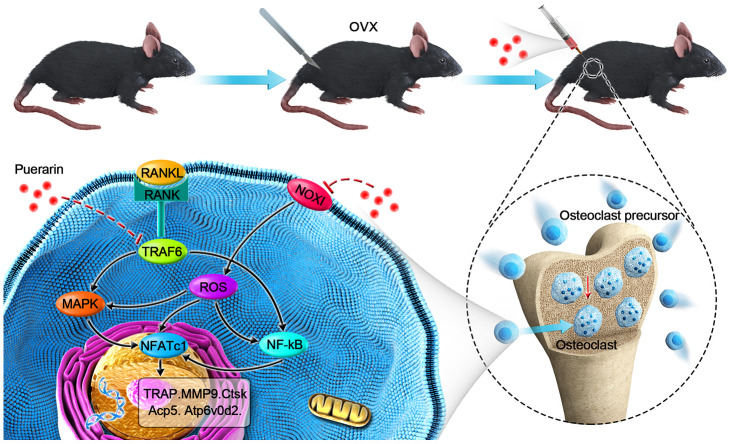
**Schematic representation of the mechanism by which puerarin inhibits *in vivo* and *in vitro* osteoclastogenesis.** RANKL induces osteoclastogenesis by binding to its receptor, RANK, thereby inducing ROS and activating the NF-κB and MAPK pathways and increased NFATc1 expression. Subsequently, the expression of osteoclast-specific genes, Ctsk, Acp5, Atp6v0d2, and Mmp9 are upregulated. Our results show that puerarin inhibits osteoclastogenesis by inhibiting intracellular ROS levels by inhibiting NOX1 and enhancing antioxidant enzymes like HO-1, subsequently inhibits the activation of the MAPK and NF-κB signaling pathways.

In conclusion, our study shows that puerarin significantly decreases OVX-induced osteoporosis by suppressing osteoclastogenesis and oxidative stress in the bone tissues. Moreover, puerarin inhibits *in vivo* and *in vitro* osteoclast differentiation and activity by decreasing ROS levels and subsequent inhibition of ROS-induced MAPK and NF-κB signaling pathways (Scheme 1). Since puerarin has minimal effects on the breast epithelial cells [66], it is a potential candidate for the treatment of osteoclast-related osteoporosis in postmenopausal women.

## MATERIALS AND METHODS

### OVX-induced osteoporosis mouse model development and puerarin treatment

The animal experiments were performed according to the protocols approved by the Institutional Animal Care Committee, Zhangjiagang Traditional Chinese Medicine Hospital (approval number: 2018A011). Briefly, we purchased eleven-week-old C57BL/6J mice (JOINN Laboratories, Suzhou, China) and randomly divided into sham, OVX and OVX+puerarin groups (6 mice per group). We prepared a 10 mM Puerarin (Sigma-Aldrich, Sydney, Australia) stock solution in dimethylsulfoxide (DMSO; Thermo Fisher Scientific, Scoresby, Australia) and diluted puerarin before use in PBS (Thermo Fisher Scientific, Scoresby, Australia). Bilateral ovariectomy was carried out to induce osteoporosis under anesthesia for the mice in OVX and OVX+Puerarin groups, a sham procedure in which the ovaries were only exteriorized but not resected was performed on sham group mice.. After one week of recovery, the mice in the OVX+puerarin group were administered with 100 mg puerarin per Kg body-weight through intraperitoneal injections every two days for six weeks before being sacrificed. The mice in the sham and OVX groups were injected intraperitoneally with 1% DMSO in PBS.

### Micro-CT scanning and analysis

The left femurs (n=6/groups) of the 3 groups of mice were analyzed using the SkyScan 1176 high-resolution micro-computed tomography scanner (SkyScan, Knotich, Belgium). The μCT images of the left femurs of the 3 groups of mice (n=6 per group) were obtained with a SkyScan 1176 high-resolution micro-computed tomography scanner (SkyScan, Knotich, Belgium). The scanning parameters were 9 μm per layer, 80 kV (voltage) and 100 mA (current). Then, the relevant 3D-images were imported after processing into the CTAn software (Bruker micro-CT, Kontich, Belgium) and the parameters such as bone mass density (BMD), bone volume/tissue volume (BV/TV), bone surface/bone volume (BS/BV), bone surface/total volume (BS/TV), trabecular number (Tb.N) and connectivity density (Conn.Dn) were measured.

### Histological and immunohistochemical analyses

After conducting the micro-CT analysis, the femurs were preserved at 4°C for histological and immunohistochemical analyses. The right femurs of the 3 groups of mice were first decalcified in 15% ethylenediaminetetraacetic acid (EDTA, Sigma), embedded in paraffin blocks, and sectioned at a thickness of 5 μm using a microtome. Then, the sections were subjected to hematoxylin and eosin (H&E), tartrate-resistant acid phosphatase (TRAcP) and immunohistochemical (IHC) staining. The primary antibodies against MMP9 (ab38898, Abcam), NFATc1 (ab25916, Abcam), Heme Oxygenase 1 (HO-1; ab13243, Abcam) and NOX1 (ab55831, Abcam) were used for immunohistochemistry. The stained sections were photographed using a Optical Microscope Olympus CX43 (Tokyo, Japan). The histomorphometric analyses of the bones were performed using the BIOQUANT OSTEO software (Bioquant Image Analysis Corporation, Nashville, TN, USA).

### *in vivo* ROS detection by DHE staining

For *in vivo* ROS detection using dihydroethidium (DHE), the bone tissues were fixed in 4% paraformaldehyde for 4 h, and decalcified in EDTA overnight. Then, the samples were incubated in 20% sucrose and polyvinylpyrrolidone (Sigma, Australia) for 24 h, embedded and frozen in gelatin. The frozen bone tissues were then cryo-sectioned at a thickness of 5 μm and air-dried before permeabilization in 0.3% Triton X-100 (Sigma, Australia) for 10 min. The nuclei were stained with DAPI. The stained sections were imaged using a Fluorescence Microscope Olympus CKX53 (Tokyo, Japan).

### *in vitro* culture and differentiation of bone marrow macrophages (BMM)

Fresh bone marrow macrophages (BMMs) were isolated from the femur and tibia of the female C57BL/6J mice. Briefly, total bone marrow cells were cultured in α-MEM medium (GE Healthcare, Pittsburgh, USA) containing 10% FBS (Gibco, Rockville, USA) and 50 ng/mL M-CSF for 24 h. Then, the non-adherent BMMs were induced with 50 ng/mL M-CSF (R&D Systems, Minneapolis, MN, USA) and 50 ng/mL RANKL (R&D Systems, Minneapolis, MN, USA). The BMMs were treated with 0, 10 and 100 μM puerarin. The concentration of DMSO in the working solution was less than 0.1%. The culture medium of the BMMs was replaced every two days.

### Cell counting kit-8 assay

The BMMs were seeded in 96-well plates at a density of 1 × 10^4^/well for 24 h. The BMMs were then treated with 0, 10, 50, 100, or 500 μM puerarin for 1, 3, or 5 days. Then, we incubated each well with 10 μl CCK-8 reagent (Beyotime, Shanghai, China) for 1-2 h. Then, the optical density (OD) was measured at 450 nm using a microplate reader (BioTek, USA).

### In vitro TRAcP staining

The BMMs were seeded in the 24-well plates (1 × 10^5^/well), incubated with 50 ng/mL M-CSF, and 50 ng/mL RANKL and treated with 0, 10 or 100 μM puerarin for 3 days. Then, the cells were washed twice for 15 min with PBS, fixed with paraformaldehyde, and analyzed using a TRAcP staining kit (Sigma, USA) according to the manufacturer’s instructions. The osteoclasts (cells with more than three nuclei) were quantified using the Image J software (NIH, Bethesda, Maryland, USA).

### Hydroxyapatite resorption assay

We cultured 1 × 10^5^ BMMs per well in 24-well hydroxyapatite-coated plates (Corning Life Sciences, St. Lowell, MA, USA) and treated with 0, 10, or 100 μM puerarin in α-MEM medium containing with 50 ng/mL RANKL and 50 ng/mL M-CSF for 5 days. Then, the wells were washed with PBS to remove cells, and the areas of hydroxyapatite resorption was observed by a Optical Microscope Olympus CKX53 (Tokyo, Japan) and quantified with the Image J software (NIH, Bethesda, Maryland, USA).

### Immunofluorescence staining

For immunofluorescence staining, the differentiated osteoclasts were washed with PBS thrice and fixed with 4% paraformaldehyde for 30 mins and then permeabilized with Triton X-100 for 10 minutes. The cells were stained with the primary antibodies against MMP9 (1:1000, ab38898, Abcam), NFATc1 (1:1000, ab25916, Abcam) at 4°C overnight, then, washed and incubated with secondary Alexa Fluor 555 antibodies (1:1000) and Molecular Probes Alexa Fluor 488 Phalloidin (Cell Signaling Technology, Danvers, USA) for 1 h in dark. After the cells were stained with DAPI for 10 minutes, The cells were imaged using a fluorescence microscope. The osteoclast area and the number of nuclei per osteoclast were quantified using the Image J software.

### Flow cytometry analysis of intracellular ROS levels

The RAW264.7 cells were stimulated with 50 ng/mL RANKL and 0, 10, or 100 μM puerarin for 30 mins. Then, the cells were incubated with α-MEM medium containing 10 μM DCFH2-DA (Beyotime, Jiangsu, China) for 30 mins at 37°C in the dark according to the manufacturer's instructions. Then, after washing the cells thrice with α-MEM, the cells were analyzed by flow cytometry (CytoFLEX Beckman, USA) and the proportions of positive cells in each group were calculated.

### Quantitative Real-time RT-PCR analysis

The RAW264.7 cells were purchased from the Cell Bank of the Chinese Academy of Sciences (Shanghai, China) and cultured in 6-well plates at a density of 1 × 10^5^/well with medium containing 50ng/mL RANKL and 0, 10, or 100 μM puerarin until osteoclasts were formed. Then total RNA was extracted from the osteoclasts using TRIzol (Ambion, USA). Equal amounts of total RNA was used for cDNA synthesis using a Reverse Transcription kit (Abm, Cat#G490 Canada). Then, real time quantitative PCR was performed using the SYBR Green PCR MasterMix (appliedbiosystems Vilnius, Lithuania). The PCR cycling parameters were as follows: 94°C for 10 min, followed by 40 cycles of 95°C for 15 s and 60°C for 60 s. The primers (Sangon Biotech, Shanghai, China) used for qPCR were as follows: NFATc1-forward:5’-CAACGCCCTGACCACCGATAG-3’; NFATc1-reverse:5’-GGCTGCCTTCCGTCTCATAGT-3’; MMP9-forward: 5’-CGTGTCTGGAGATTCGACTTGA-3’, MMP9-reverse: 5’-TTGGAAACTCACACGCCAGA-3’, Ctsk-forward: 5’-GGGAGAAAAACCTGAAGC-3’, Ctsk-reverse: 5’-ATTCTGGGGACTCAGAGC-3’, Acp5-forward: 5’-TGTGGCCATCTTTATGCT-3’, Acp5-reverse: 5’-GTCATTTCTTTGGGGCTT-3’, c-Fos-forward: 5’-GCGAGCAACTGAGAAGAC-3’, c-Fos-reverse: 5’-TTGAAACCCGAGAACATC-3’) and GAPDH-forward: 5’-GGTGAAGGTCGGTGTGAACG-3’, GAPDH-reverse: 5’-CTCGCTCCTGGAAGATGGTG-3’. Each sample was tested three times to ensure accuracy. The expression of NFATc1, MMP9, CTSK, Acp5 and cFos mRNAs was measured relative to GAPDH mRNA using the 2^-ΔΔCt^ method.

### Lentiviral packaging and transfections

The lentiviruses carrying vectors with the mouse HO-1 and Traf6 genes were constructed and produced by OBiO Technology (Shanghai, China). The RAW264.7 cells were infected with the pSLenti-EF1a-EGFP-P2APuro-CMVTraf6-3Flag and pLKD-CMVEGFP-2A-Puro-U6-shRNA (Hmox1) at a MOI of 100 for 72 h following which stably transfected cell lines were obtained by culturing in medium containing puromycin according to the manufacturer's instructions.

### Western blotting

The RAW264.7 cells were cultured in 6-well plates at a density of 1 × 10^5^/well and induced with 50 ng/mL RANKL in the presence or absence of different puerarin concentrations for different time lengths, as stated. Then, the total protein samples were extracted using RIPA buffer and quantified using the BCA protein assay. Equal amounts of protein samples were resolved on a 15% sodium dodecyl sulfate-polyacrylamide gel electrophoresis (SDS-PAGE) for 2 h and transferred onto a polyvinylidene fluoride (PVDF) membrane (Merck Millipore, USA). The membranes were blocked with 5% skimmed milk for 60 mins at room temperature. Then, the membranes were incubated overnight at 4°C with primary antibodies against NOX1 (1:1000, ab55831), NOX2 (1:1000, ab80508), NOX4 (1:1000, ab133303), TNF receptor associated factor 6 or TRAF6 (1:1000, ab227560), Catalase (1:500, ab76110), Heme Oxygenase 1 (HO-1 1:2000, ab13243), c-Fos, MMP9, CTSK, IκB-α (1:1000, ab32518), NF-κB (1:1000, ab16502), JNK (1:1000, ab179461), p-JNK (1:1000, ab32503), and p38 (1:1000, ab170099), which were all purchased from Abcam. The primary antibodies against ERK (1:1000, #4695), p-ERK (1:1000, #4377), p-IκB-α (1:1000, #2859), p-NF-κB (1:1000, #3031), p-p38 (1:1000, #4511), p-JNK (1:1000, #4668), and GAPDH (1:1000, #51332) were purchased from Cell Signaling Technology (CST). Then, the membranes were rinsed in Tris-buffered saline with Tween 20 (TBST) and incubated with the corresponding horseradish peroxidase-conjugated secondary antibodies (1:1000) for 2 h at room temperature. The blots were then developed using ECL (Millipore Corporation, Billerica, MA, USA) and then imaged and quantified using the Image J software.

### Statistical analysis

SPSS 25 software was used for statistical analysis in this experiment. All experiments were repeated at least three times independently. The data are shown as the means ± standard deviation (SD), where SD denotes the differences between three independent experimental values. The data with normal distribution was analyzed using parametric tests and D'Agostino-Pearson omnibus test. For comparisons between multiple samples, one-way ANOVA and Tukey's test was used. *P <*0.05 was considered statistically significant.

## Supplementary Material

Supplementary Methods

Supplementary Figures
