# Supplementary material for: Puerarin alleviates osteoporosis in the ovariectomy-induced mice by suppressing osteoclastogenesis via inhibition of TRAF6/ROS-dependent MAPK/NF-κB signaling pathways
Source: Aging (Albany NY). 2020 Nov 7;12(21):21706–29. doi: 10.18632/aging.103976 (PMC7695364; doi:10.18632/aging.103976)
Supplement: Supplementary Methods [file aging-12-103976-s001..pdf]

Supplementary Methods

Cell culture

MC3T3-E1 cells were cultured in α-MEM (HyClone, Logan, UT, USA) medium containing 10% fetal bovine serum and 1% penicillin-streptomycin at 5% CO_2 and 37˚C. To induce the differentiation of MC3T3-E1 cells, the cells were cultured in osteogenic differentiation medium that included Dulbecco's modified Eagle medium (DMEM) containing 10% bovine serum, 100 µg/mL streptomycin, 100 U/mL penicillin, 50 µg/mL L-ascorbic acid, 100 nM dexamethasone, and 10 mM β-glycerol phosphate. We purchased hydrogen peroxide (H_2O_2) and Puerarin from Sigma (St. Louis, MO, USA). For treatments, the cells were classified into four experimental groups: (1) control group: cells treated only with α-MEM medium; (2) H_2O_2 group: cells treated with α-MEM containing 400 µM H_2O_2; (3) low-dose puerarin group: cells treated with α-MEM containing 400 µM H_2O_2 and 10 µM puerarin; and (4) high-dose puerarin group: cells treated with α-MEM containing 400 µM H_2O_2 and 100 µM puerarin.

ALP staining

After two weeks of culture in osteogenic medium, MC3T3-E1 cells were assayed for alkaline phosphatase (ALP) activity. Briefly, the MC3T3-E1 cells were washed thrice with PBS and fixed with 4% paraformaldehyde for 15 mins. Then, the cells were washed thrice with PBS and incubated with the BCIP/NBT working solution (Beyotime Biotech, Jiangsu, China) in the dark for 20 mins. The staining was observed and photographed under a light microscope.

Alizarin red S staining

After three weeks of culture in osteogenic medium, MC3T3-E1 cells were stained with Alizarin red S (ARS). Briefly, the MC3T3-E1 cells were washed three times with PBS and fixed with 4% paraformaldehyde for 20 min at 4 °C. Then, the cells were rinsed and incubated with the ARS staining solution (pH 4.2; Cyagen Biosciences, Santa Clara, CA, USA) for 20 min. Finally, the cells were washed in ddH2O thrice and imaged under a light microscope.

MDC staining

To determine the presence of autophagic vesicles in the osteoclast cells, RAW264.7 cells were grown on the cover slips in a 6-well plate and induced to differentiate into osteoclasts with or without RANKL in the presence or absence of puerarin and H_2O_2. During the course of differentiation, a subset of cells on day 2 were incubated with 50 mM MDC (Beyotime Biotech, Jiangsu, China) at 37°C for 15 min and then washed with PBS. Finally, the cells were mounted on a glass slide and imaged using a fluorescence microscope.
