# Supplementary material for: Puerarin alleviates osteoporosis in the ovariectomy-induced mice by suppressing osteoclastogenesis via inhibition of TRAF6/ROS-dependent MAPK/NF-κB signaling pathways
Source: Aging (Albany NY). 2020 Nov 7;12(21):21706–29. doi: 10.18632/aging.103976 (PMC7695364; doi:10.18632/aging.103976)
Supplement: Supplementary Figures [file aging-12-103976-s002..pdf]

Supplementary Figures

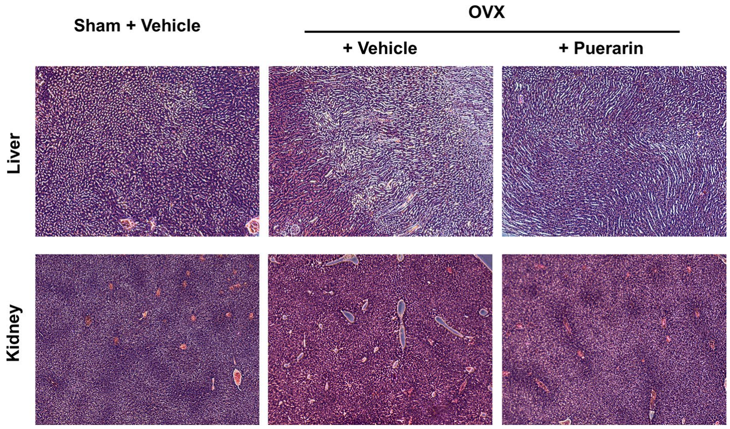

Supplementary Figure 1. H&E staining of the liver and kidney tissue sections in the puerarin treated OVX-induced osteoporosis model mice.

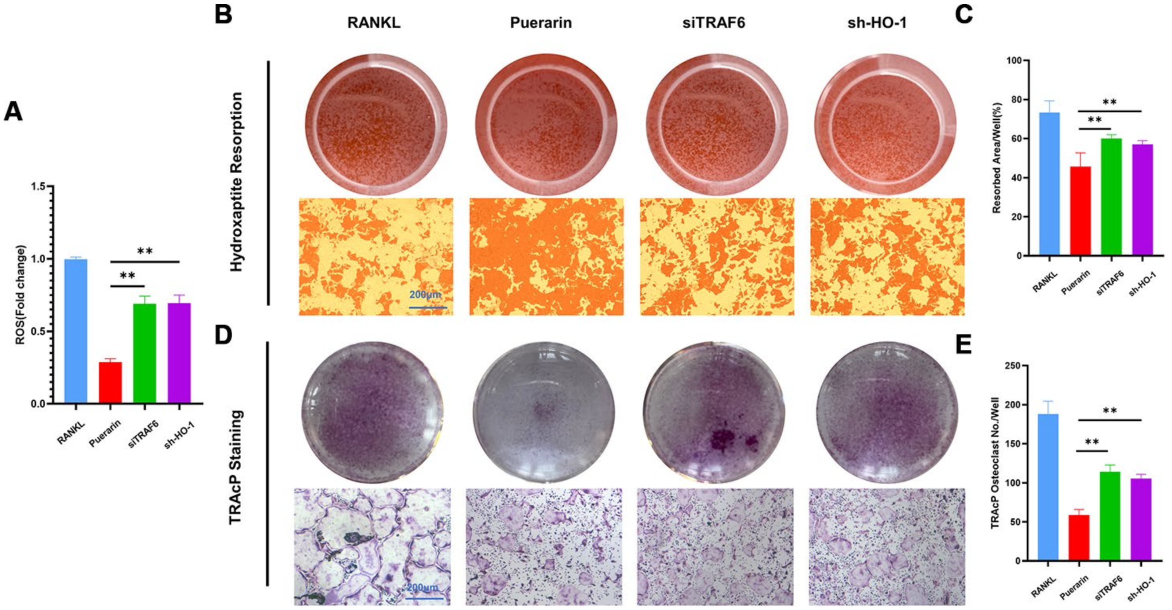

Supplementary Figure 2. Puerarin inhibits *in vitro* hydroxyapatite resorption activity of the osteoclasts and *in vitro* osteoclastogenesis by upregulating HO-1 and downregulating TRAF6. (**A**) Flow cytometry analysis of ROS levels by DCFH2-DA staining of control, HO-1 knockdown or TRAF6 overexpressing RAW264.7 cells treated with RANKL+puerarin. Note: n=3 per group, ** *P* <0.01 vs. the Puerarin-treatment control group. (**B**) Representative images show osteoclastogenesis efficiency based on hydroxyapatite resorption assay in the control, HO-1 knockdown or TRAF6 overexpressing RAW264.7 cells treated with RANKL+puerarin. (**C**) Quantitative analysis shows the hydroxyapatite resorbed area per osteoclast and the total resorbed hydroxyapatite area in the control, HO-1 knockdown or TRAF6 overexpressing RAW264.7 cells treated with RANKL+puerarin. Note: n=3 per group; ***P* <0.01 vs. the Puerarin-treatment group. (**D**) Representative images show osteoclastogenesis efficiency based on TRAcP staining in the control, HO-1 knockdown or TRAF6 overexpressing RAW264.7 cells treated with RANKL+puerarin. (**E**) Quantitative analysis of the TRAcP staining assay results show of the numbers of TRAcP-positive cells (>3 nuclei) in the control, HO-1 knockdown or TRAF6 overexpressing RAW264.7 cells treated with RANKL+puerarin. Note: n=3 per group; ***P* <0.01 vs. the Puerarin-treatment group.

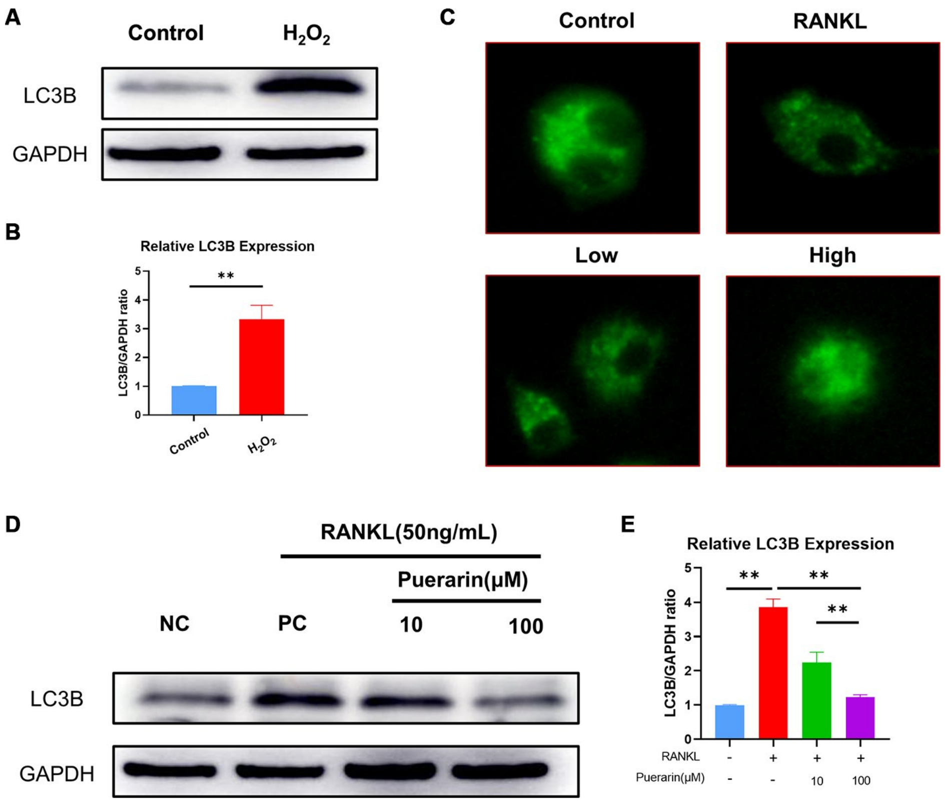

Supplementary Figure 3. Puerarin suppresses autophagy in the *in vitro* differentiated osteoclasts. (**A**, **B**) Western blot analysis shows LC3B levels in the control, H_2O_2-induced RAW264.7 cells. Note: n=3 per group; ** *P* <0.01 vs. the control group. (**C**) Representative images show the MDC staining in the control, RANKL-induced and RANKL+Low/High dose puerarin-treated RAW264.7 cells. (**D**–**E**) Western blot analysis of cell lysates with antibodies against LC3B in the control, RANKL-, low-dose puerarin+ RANKL-, and high-dose puerarin+ RANKL-treated RAW264.7 cells. Note: n=3 per group, ** *P* <0.01 vs. the RANKL-induced group.

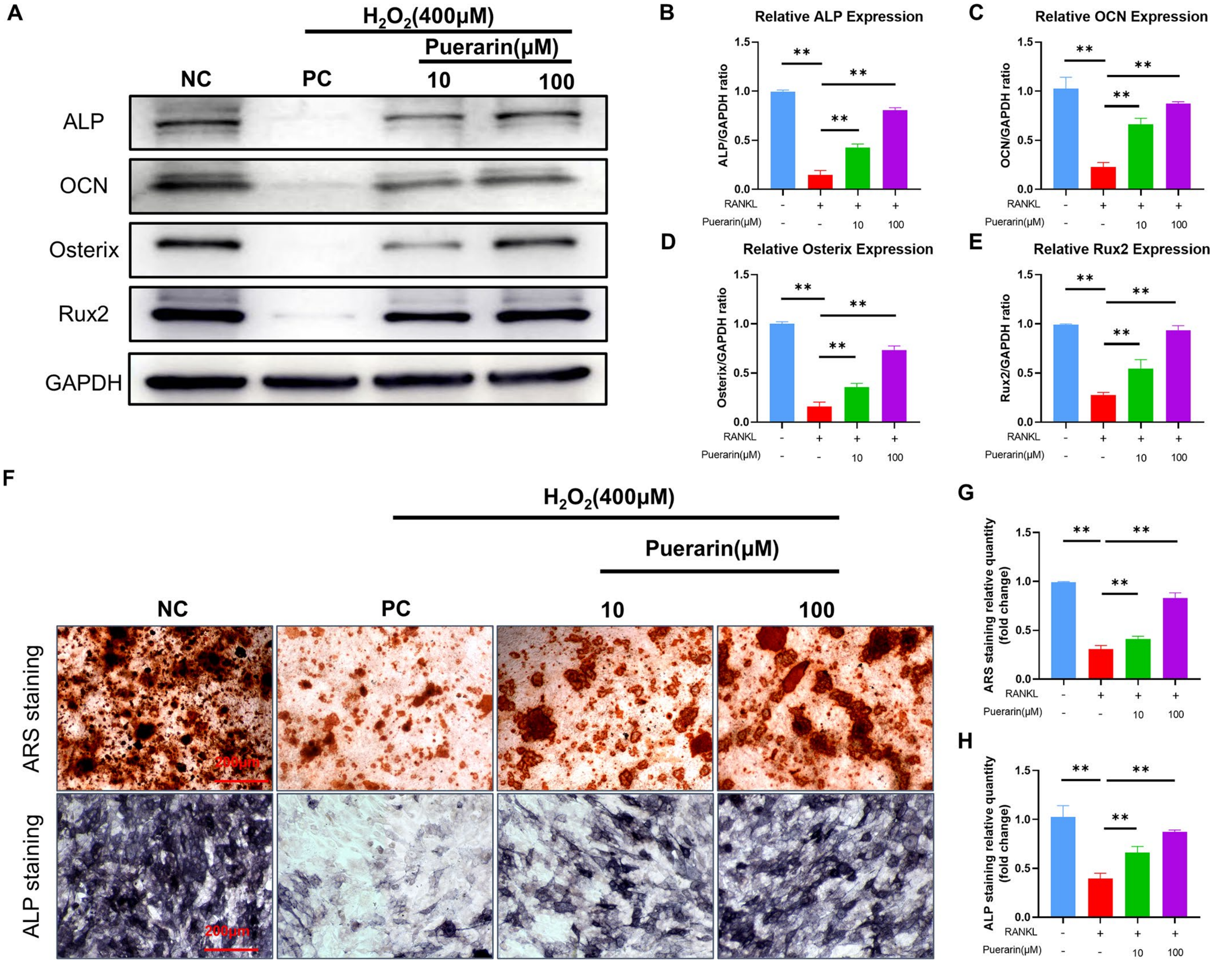

Supplementary Figure 4. Puerarin reduces ROS-induced inhibition of osteoblast differentiation in MC3T3-E1 cells. (**A**–**E**) Western blot analysis shows the expression of ALP, OCN, Osterix and Rux2 proteins in the control, H_2O_2-, low-dose puerarin+ H_2O_2-, and high-dose puerarin+ H_2O_2-treated MC3T3-E1 cells. Note: n=3 per group, ** *P* <0.01 vs. the H_2O_2-induced group (**F**–**H**) Representative images and quantitative analysis shows the ARS and ALP staining in the control, H_2O_2-, low-dose puerarin+ H_2O_2-, and high-dose puerarin+ H_2O_2-treated MC3T3-E1 cells. Note: n=3 per group, ** *P* <0.01 vs. the H_2O_2-induced group.

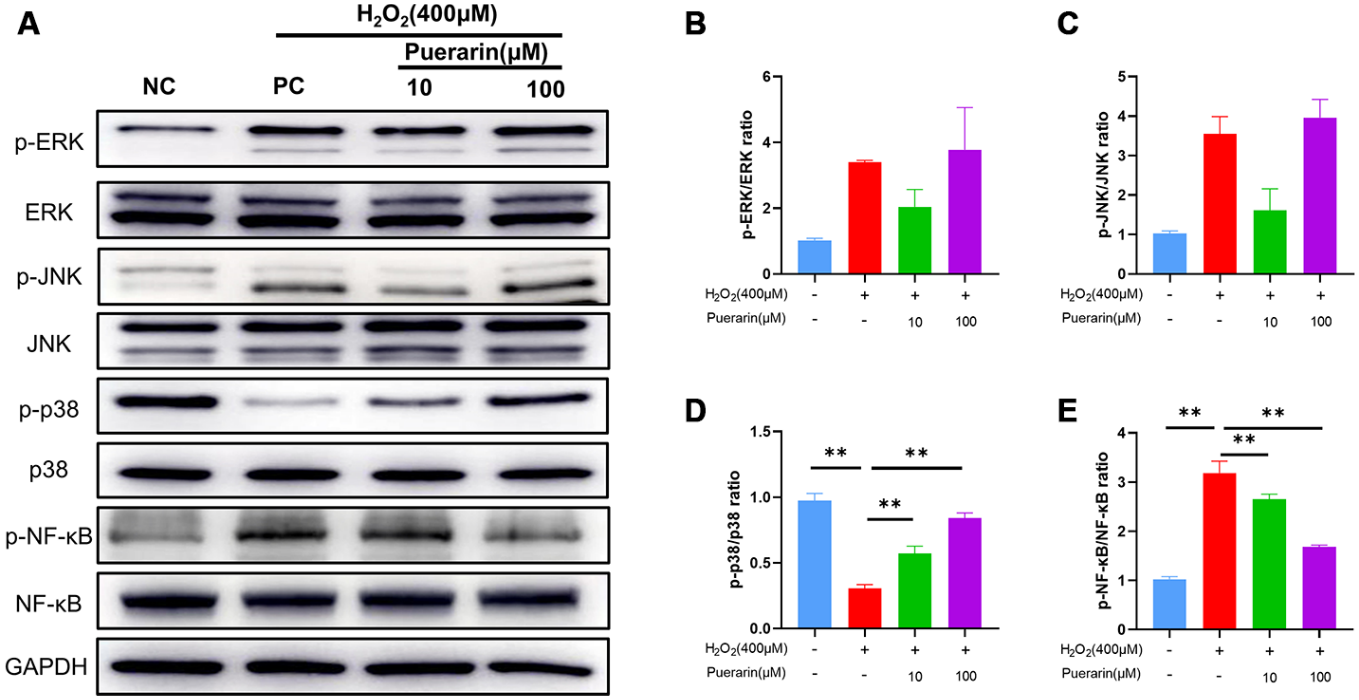

Supplementary Figure 5. Puerarin reduces ROS-induced inhibition of osteoblast differentiation by activating p38 MAPK and downregulating NF-κB signaling. (**A**) Western blot analysis shows the levels of p-ERK, ERK, p-JNK, JNK, p-p38, p38, p-NF-κB, NF-κB and GAPDH in the control, H_2O_2-, low-dose puerarin+ H_2O_2-, and high-dose puerarin+ H_2O_2-treated MC3T3-E1 cells. (**B**–**E**) Quantitative analysis shows ratios of p-ERK/ERK, p-JNK/JNK, p-p38/p38 and p-NF-κB/NF-κB in the control, H_2O_2-, low-dose puerarin+ H_2O_2-, and high-dose puerarin+ H_2O_2-treated MC3T3-E1 cells. Note: n=3 per group; ** *P* <0.01.
